# The Antiaging Properties of *Andrographis paniculata* by Activation Epidermal Cell Stemness

**DOI:** 10.3390/molecules200917557

**Published:** 2015-09-22

**Authors:** Jiyoung You, Kyung-Baeg Roh, Zidan Li, Guangrong Liu, Jian Tang, Seoungwoo Shin, Deokhoon Park, Eunsun Jung

**Affiliations:** 1 Biospectrum Life Science Institute, Sangdaewon-Dong, Seongnam-City, Gyeonggi-Do 442-13, Korea; E-Mails: biotr@biospectrum.com (J.Y.); biosh@biospectrum.com (K.-B.R.); biost@biospectrum.com (S.S.); pdh@biospectrum.com (D.P.); 2 Infinitus, Rm 102 PCI Business Building, No. 66 Jiangzhong Road, Tianhe District, Guangzhou 510665, China; E-Mails: Sunny.Zidan.Li@infinitus-int.com (Z.L.); Jim.Liu@infinitus-int.com (G.L.); Tom.Tang@infinitus-int.com (J.T.)

**Keywords:** *Andrographis paniculata*, antiaging, epidermal stemness, integrin β1

## Abstract

*Andrographis paniculata* (*A. paniculata*, *Chuanxinlian*), a medicinal herb with an extremely bitter taste that is native to China and other parts of Southeast Asia, possesses immense therapeutic value; however, its therapeutic properties have rarely been applied in the field of skin care. In this study, we investigated the effect of an *A. paniculata* extract (APE) on human epidermal stem cells (EpSCs), and confirmed its anti-aging effect through *in vitro*, *ex vivo*, and *in vivo* study. An MTT assay was used to determine cell proliferation. A flow cytometric analysis, with propidium iodide, was used to evaluate the cell cycle. The expression of integrin β1 (CD29), the stem cell marker, was detected with antibodies, using flow cytometry *in vitro*, and immunohistochemical assays in *ex vivo*. Type 1 collagen and VEGF (vascular endothelial growth factor) were measured using an enzyme-linked immunosorbent assay (ELISA). During the clinical study, skin hydration, elasticity, wrinkling, sagging, and dermal density were evaluated before treatment and at four and eight weeks after the treatment with the test product (containing the APE) on the face. The proliferation of the EpSCs, treated with the APE, increased significantly. In the cell cycle analysis, the APE increased the G2/M and S stages in a dose-dependent manner. The expression of integrin β1, which is related to epidermal progenitor cell expansion, was up-regulated in the APE-treated EpSCs and skin explants. In addition, the production of VEGF in the EpSCs increased significantly in response to the APE treatment. Consistent with these results, the VEGF and APE-treated EpSCs conditioned medium enhanced the Type 1 collagen production in normal human fibroblasts (NHFs). In the clinical study, the APE improved skin hydration, dermal density, wrinkling, and sagging significantly. Our findings revealed that the APE promotes a proliferation of EpSCs, through the up-regulation of the integrin β1 and VEGF expression. The VEGF might affect the collagen synthesis of NHF as a paracrine factor. Clinical studies further suggested that treatment with formulations containing APE confers anti-aging benefits. Based on these results, we suggest that APE may be introduced as a possible anti-aging agent.

## 1. Introduction

*Andrographis paniculata* (Burm.f.) Nees *(Acanthaceae)*, known generally as the “king of bitters”, is widely used in China, India, and other Southeast Asian countries [1]. This plant has been applied for treating sore throat, flu, and upper respiratory tract infections. Its major active constituent, andrographolide, exhibits a broad range of biological properties, such as anti-inflammatory, antibacterial, antitumor, antidiabetic, antimalarial, and hepatoprotective [2,3].

In regard to human skin, aging is associated with a loss of fibrous tissue, a slower rate of cellular renewal, and a reduced vascular and glandular network; the barrier function that maintains cellular hydration also becomes impaired. The epidermis is a multilayered epithelium that protects the skin from environmental assault and damage. The hallmark of the epidermis is its ability to self-renew throughout the entire life span of the organism; this is achieved with the epidermal stem cells (EpSCs) in the basal cell layer of the epidermis, which undergo an unlimited number of symmetric and asymmetric cell divisions, giving rise to daughter cells that either proliferate or exit the cell cycle, and move to the suprabasal layer of the epidermis. Several studies have reported that skin aging is associated with a reduced expression of stem cell markers such as p63 and integrin β1 [4,5]. The expression of integrin β1 is critically important for the expansion of the epidermal progenitor cells, in order to maintain epidermal homeostasis. The reduction in the integrin β1 levels, with advancing age, contributes to the age-associated changes in dermal thickness and skin vascularization; therefore, aged human epidermal cells exhibit a reduced *in vitro* self-renewal ability.

Along with the identification of the molecular mechanisms of the epidermal cells, stemness signaling points to a need for new therapeutic alternatives to treat and prevent skin aging. Until now, no studies have evaluated the influence of an APE on skin aging. As such, this study was conducted to evaluate the effects of an APE on epidermal cell stemness, and the possible mechanisms underlying these effects *in vitro*. We also confirmed the anti-aging effect of APE in clinical study.

## 2. Results and Discussion

### 2.1. APE Induces EpSCs Proliferation

**Figure 1:**
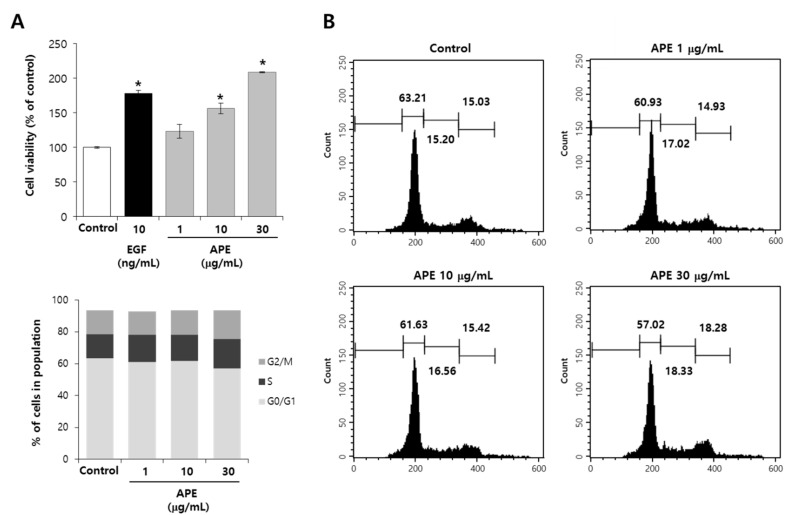
APE induced proliferation of EpSCs. (**A**) The EpSCs were cultured in a serum-free condition, with the APE, for three days; the proliferation was measured by MTT; (**B**) Under the same condition for 24 h, the cell cycle distribution was evaluated by using a flow cytometry analysis with PI staining. Indicated values are suggested as 100% of the control (*n* = 3). The data are presented as the mean ± SD, * *p* < 0.005.

In aged epidermis, there is a progressive decrease in the renewal rate of epidermal cells result from decreased proliferation [6] and senescent keratinocytes [7]. Reduced proliferation of the epidermal cells in aging skin causes a disruption in the permeability of the skin, and a distortion of enzyme activity that consequently leads to the loss of hydration and epidermal aging. Epidermal stem cells reside in a unique niche within the skin, playing a crucial role in self-renewal of the epidermis. To investigate whether APEs could induce the proliferation of EpSCs, we treated the EpSCs with the APE, and verified the increased EpSCs proliferation initially. As shown in Figure 1A, the EpSCs proliferation following the APE treatment at 1, 10, and 30 μg/mL was increased to the level of 23%, 56%, and 108%, respectively.

### 2.2. APE Induces Cell Cycle Progression

To confirm the proliferative effect of the APE on the EpSCs, we examined the cell cycle distribution by conducting a flow cytometry analysis with PI. As shown in Figure 1B, the APE appeared to stimulate cell cycle progression in the EpSCs. The G0/G1 phase was decreased through treatment with any concentration of APE, compared to the untreated control, while the S phase and G2/M phase was increased through treatment with the APE. These results indicated that the APE had the capacity to increase the EpSCs’ proliferation.

### 2.3. APE Increased Integrin β1 (CD29) Expression in Vitro and ex Vivo

**Figure 2:**
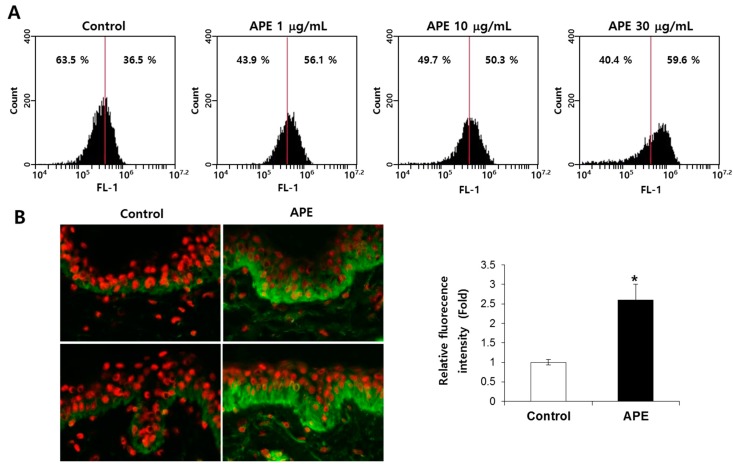
APE increased integrin β1 expression *in vitro* and *ex vivo*. (**A**) A FACS analysis of the integrin β1 expression in the EpSCs’ integrin β1 antibody-conjugated cells shift to the right panel compared to the control. (**B**) The explants were kept in survival in a BEM culture medium for 10 days with the vehicle and 0.1% APE; integrin β1 (CD29) immunostaining was conducted. (*n* = 3). The data are presented as the mean ± SD, * *p* < 0.005.

In the skin, integrin β1 plays a role in epidermal stem cell maintenance and proliferation, and is considered to be a marker of epidermal stem cells. Among the high proliferative keratinocytes, integrin β1 expressed higher than the other epidermal cells [8]. In mice, a deficiency of integrin β1 induced a epidermal proliferation disorder (like basement membrane and hair follicle formations based on hemidesmosome stability) *in vivo* [9]. Additionally, integrin β1 contributes to the survival of human adult epithelial progenitor cells (ePCs), enhancing the outer root sheath keratinocytes in hair follicles [10]. To confirm the involvement of integrin β1 in the EpSCs’ proliferation, we examined the integrin β1 expression of the EpSCs through a flow cytometry analysis. The cell surface expression of integrin β1 significantly increased following the APE treatment, in a dose-dependent manner (Figure 2A). Additionally, we confirmed that the APE dramatically increased the expression of integrin β1 in the human skin explant (Figure 2B). Based on these results, we suggest that the APE-induced pro-proliferation of the EpSCs was promoted by the activation of integrin β1.

### 2.4. APE Up-Regulates the VEGF Secretion from the EpSCs

**Figure 3:**
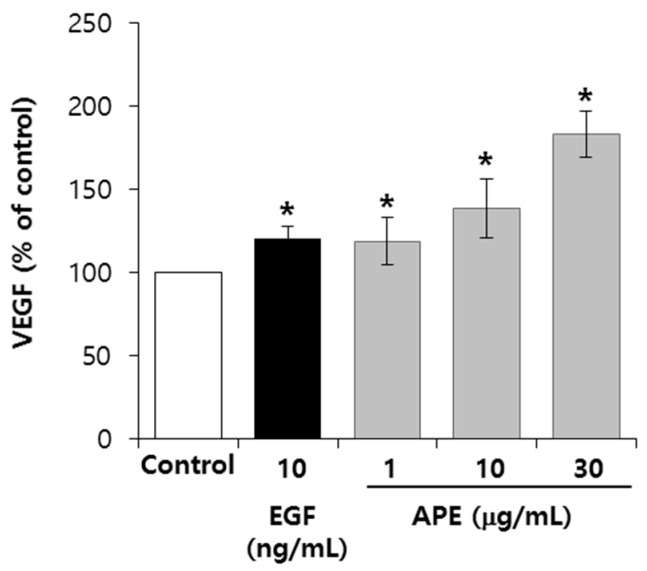
APE upregulates the VEGF secretion from the EpSCs. The EpSCs were cultured in a serum-free condition, with or without APE, for three days. Culture supernatants from the EpSCs were added to 96 well plates, coated with a polyclonal antibody of VEGF, and then sequentially proceeded using the streptavidin-biotin detection method. (*n* = 3). The data are presented as the mean ± SD, * *p* < 0.005.

Keratinocytes secrete a variety of cytokines, such as the transforming growth factor β (TGF-β), the fibroblast growth factor 2 (FGF-2), the epidermal growth factor (EGF), interleukin-6 (IL-6), and the vascular endothelial growth factor (VEGF); these factors affect the proliferation and stemness of the EpSCs. To confirm the involvement of growth factors and cytokine on the EpSCs proliferation, we examined the production of cytokines in EpSCs, using the ELISA kit. As shown in Figure 3, the VEGF production following the treatment of APE at 1, 10, and 30 μg/mL increased to the levels of 14%, 32%, and 74%, respectively. The APE showed no significant changes in other cytokines, including FGF-2, EGF, HGF, and IL-6 (data not shown). Several studies have reported the relationship between integrin β1 and VEGF. During the process of wound healing, integrin β1 is required for angiogenesis in the epidermis, with α3. In addition, VEGF drives integrin β1 to support endothelial cell (EC) proliferation or migration through ERK1/2, a mitogen-activated protein kinase (MAPK) signaling [11,12].

### 2.5. VEGF and APE Treated EpSCs Conditioned Medium Increases Type 1 Collagen Production in NHFs

**Figure 4:**
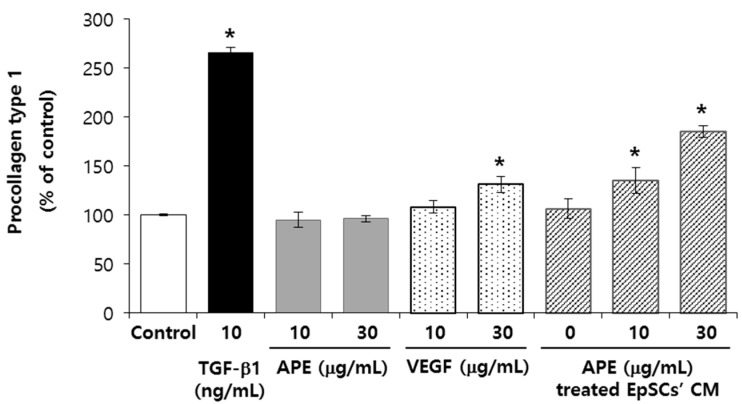
The VEGF and APE treated EpSCs’ conditioned medium increase the type 1 collagen production in NHFs. The NHFs were cultured in a serum-free condition with APE and VEGF, and incubated for two days. The EpSCs’ conditioned medium (CM), with or without the APE treatment, was collected after two days of being cultured. Then, NHFs were incubated in the CM for two days. To detect procollagen Type 1 in the NHFs, culture supernatants with antibody-POD conjugate solution from ELISA kit were used. (*n* = 3). The data are presented as the mean ± SD, * *p* < 0.005.

Epidermal keratinocytes release cytokines that indirectly affect the extra cellular matrix (ECM) production in dermal fibroblasts [13]. VEGF increases collagen synthesis via MAPK signaling pathway in human mesangial cells [14], but the role of VEGF on collagen synthesis in dermal fibroblasts remains to be elucidated [15]. To explore the mechanism of the effects of the APE on dermal aging, we investigated the paracrine effects of the APE-treated EpSCs on human dermal fibroblasts. The APE did not show any significant effect on collagen synthesis in NHFs, whereas the APE-treated EpSCs’ conditioned medium increased collagen synthesis up to 185%, at 30 μg/mL (Figure 4). VEGF, which are released from the EpSCs by the APE, also stimulated collagen synthesis. Taken together, these findings suggest that the stimulatory effect of the APE on the collagen synthesis of NHFs may be mediated by the VEGF.

### 2.6. APE Show Anti-Aging Properties in Vivo

**Figure 5:**
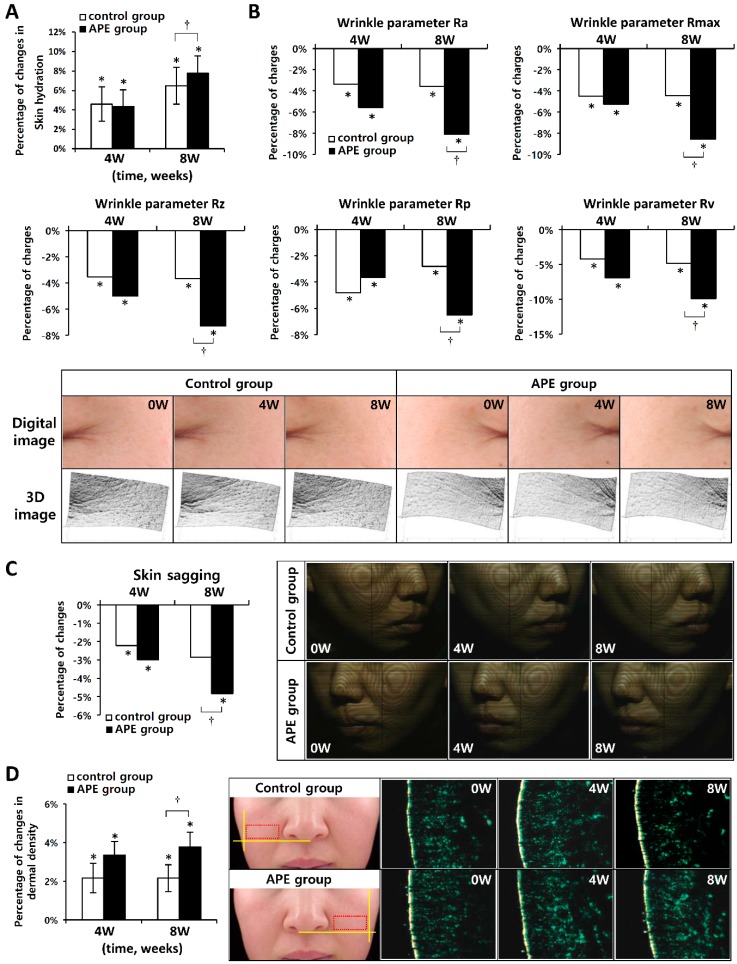
Clinical trial changes in skin hydration, wrinkling, sagging and dermal density, following eight consecutive weeks of test product application, respectively, were observed and monitored. (**A**) Skin hydration was detected using a capacitance method; (**B**) The decrement rate of the wrinkle parameters: Ra, Rmax, Rz, Rv, Rp. Digital and 3D imaging indicated a difference in wrinkle values; (**C**) Skin sagging was detected using contour line imaging and the decrement rate; (**D**) A dermal density analysis was confirmed using an ultrasonic system. Mean ± SEM. * *p* < 0.05 *vs*. before treatment, ^†^ *p* < 0.05 *vs.* Control group.

To check whether the APE has an anti-aging effect on human skin, we performed clinical tests concerning skin hydration, wrinkling, sagging, and dermal density. 32 female subjects, aged between 40 and 51 (average age: 47.09 ± 2.73 year), participated in the study. After eight weeks of topical application, both groups showed an increase in skin hydration (6.49% for the control group, and 7.77% for the APE group, respectively); skin hydration was significantly high, at eight week, in the APE group (*p* < 0.05) (Figure 5A).

We analyzed skin wrinkling using 3D imaging. Both groups showed significantly improved skin wrinkling at four and eight weeks after the treatment, compared to before the treatment. In the APE treated group, all parameters (Ra, Rmax, Rz, Rp, and Rv) were significantly decreased compared to the control group (*p* < 0.05) (Figure 5B).

Contour line imaging was used to measure the skin sagging level. After eight weeks of topical application, both groups showed a decrease in skin sagging (2.8% for the control group, 4.68% for the APE group). Skin sagging was significantly low, at eight weeks after treatment, in the APE group (*p* < 0.05) (Figure 5C).

An ultrasonic system was used to measure changes in the dermal density. The increment rates of the control group were 2.15% and 2.17%, and the increment rates of the APE group were 3.36% and 3.81%, respectively (Figure 5D), at four and eight weeks after treatment. The dermal density was significantly higher, at eight weeks after treatment, in the APE group compared to the control group (*p* < 0.05) (Figure 5E).

This clinical trial confirmed that the topical application of APEs, over a period of eight weeks, can significantly improve skin hydration, wrinkling, sagging (lifting), and dermal density compared to using the vehicle alone.

## 3. Experimental Section

### 3.1. Cell Culture

Epidermal stem cells were isolated from the human foreskin, using a method from Lee *et* *al* [16]. These cells were added to a Type 4 collagen-coated dish containing a keratinocyte culture medium (EpiLife, Invitrogen, Carlsbad, CA, USA). After a 4 h incubation period, the medium containing non-adherent cells was discarded, and only the rapidly adhering cells were cultured, at 37 °C, in a 5% CO_2_ incubator. The cells were sorted using CD49f and CD29 antibodies. Normal human fibroblasts (NHFs, ATCC PCS-201-012) were purchased from ATCC (American Type Culture Collection, Manassas, VA, USA) and cultured in Dulbecco’s modified Eagle’s medium (DMEM, Hyclone, Logan, UT, USA) including 4 mM L-glutamine, 4500 mg/L glucose, sodium pyruvate supplemented with 1% penicillin and streptomycin (Gibco, Carlsbad, CA, USA), and 10% fetal bovine serum (FBS, Gibco, Carlsbad, CA, USA) at 37 °C, 5% CO_2_ incubator.

### 3.2. Preparation of the Andrographis paniculata Extract (APE)

Whole *Andrographis paniculata* bodies (300 g dry weight) were homogenized and extracted, with 50% ethanol, by sonication in an ultrasound bath for 1 h at 40 °C. The extracts were then concentrated in vacuum to yield 50 g of extract, after which they were re-dissolved with distilled water, and then partitioned by an equal volume of *n*-hexane, chloroform, ethyl acetate, and *n*-butanol. The ethyl acetate extracts were concentrated in a vacuum, from which 10 g of residue was obtained.

### 3.3. Cell Viability Assay

The general viability of the cultured cells was determined by the reduction of 3-(4,5-dimethyl-2-thiazoyl)-2,5-diphenyl-2H-tetrazolium bromide (MTT) to formazam. After the incubation of the cells treated with the APE, the medium was changed to a serum-free keratinocyte medium (EpiLife) containing MTT (0.1 mg/mL). The cells were incubated for 1 h and 30 min, at 37 °C, and solubilized with dimethyl sulfoxide (DMSO, Sigma, St. Louis, MO, USA). The absorbance was measured at 570 nm, using a spectrophotometer (Power Wave, Bio-Tek Inc., Winooski, VT, USA).

### 3.4. Cell Cycle Analysis

A flow cytometric analysis, with propidium iodide (PI), was performed in order to measure the cell cycle progression. Both the adherent and floating cells were collected, washed with ice-cold PBS, and fixed with 70% ice-cold ethanol for 1 h before repeated washing with PBS and re-suspension with Rnase A (0.5 mg/mL) in PBS. After 1 h of incubation, the cellular DNA was stained with PI (50 μg/mL) for 20 min while protected from the light at 4 °C. The relative DNA concentration of the stained cells was measured using a FACS Calibur flow cytometer (Becton Dickinson, San Jose, CA, USA).

### 3.5. Integrin β1 Flow Cytometry

The cell-surface expression of integrins can be differentiated from the intracellular pools by a flow cytometry analysis. To quantify the integrin β1 in EpSCs, the cells were harvested and analyzed according to the manufacturer’s instructions (eBioscience, San Diego, CA, USA). The cell surface was stained with integrin β1 antibody, conjugated to fluorochrome (Abcam, Cambridge, MA, USA) for 30 min on ice; the protein level was detected level using a FACS Calibur flow cytometer (BD Biosciences, San Jose, CA, USA).

### 3.6. Immunohistochemical Analysis

The skin explants, with an average diameter of 12 mm, were prepared from an abdominoplasty of a 49-year-old Caucasian woman (BIO-EC reference: P1181-AB49). The explants were kept alive in a BEM culture medium at 37 °C in a humid, 5% CO_2_ atmosphere. The APE was introduced in a carboxymethyl cellulose (CMC) gel, with a final concentration of 0.1%, and was topically applied to D0, D2, D4, D7, and D9. The immunostaining of integrin β1 was performed on frozen sections, with an anti-integrin β1 mouse monoclonal antibody clone 4B7R (Santa Cruz Biotechnology, Santa Cruz, CA, USA) at 1:300, for 2 h at room temperature, using the Vectastain Kit Vector amplifier system avidin/biotin (Vector laboratories, Burlingame, CA, USA), and was revealed using fluorescein isothiocyanate (FITC). The nuclei were post-stained with PI. The integrin β1 was observed using a fluorescence microscope (type DMLB), at the magnification of 40×; photos were taken with a numeric DP72 Olympus camera, and stored using the CellD storing software.

### 3.7. ELISA Assay

The VEGF and Type 1 procollagen concentrations in the culture supernatant from the EpSCs and NHF cells were determined using a commercially available ELISA kit, according to the manufacturer’s instructions. Culture supernatants from the EpSCs were added to 96 well plates, coated with polyclonal antibodies of the VEGF and then sequentially proceeded to the streptavidin-biotin detection method (R & D systems, Company, Minneapolis, MN, USA). To detect procollagen Type 1 in NHFs, culture supernatants with an antibody-POD conjugate solution from the ELISA kit (Takara, Otsu, Shiga, Japan) were incubated at 37 °C for 3 h. After that, they were washed four times with PBS and added substrate. The absorbance was measured at 450 nm.

### 3.8. Statistical Analysis

All experiments were conducted at least three times, and the data were expressed as the mean ± SD. Data was analyzed by student’s *t*-test. A value of *p* < 0.05 was considered to be statistically significant.

### 3.9. Clinical Anti-Aging Study

#### 3.9.1. Human Volunteers

Thirty-two healthy female Korean volunteers, aged 40–53 years (mean ± SI = 47.09 ± 2.73 years) were selected for this study. Several skin characteristics related to aging, like skin hydration, elasticity, wrinkling, sagging and dermal density were evaluated before treatment, and at four and eight weeks after treatment using test products (A group: Control Product, B group: Test Product) on the face. This study was approved by the ethics committee of the DERMAPRO/Skin Research Center (Seoul, Korea), and subjects gave written informed consent. The investigator primarily selected potential study subjects who satisfied the inclusion and exclusion criteria.

**Table 1:** Formulation of APE containing cream and placebo cream.

Phase	Ingredient	% W/W Placebo (A Group)	% W/W Test (B Group)
A	Deionized Water	To 100	To 100
	Carbomer941	0.20	0.20
	Xanthan Gum	0.05	0.05
B	HerbEx Hyaluron 1.0	1.00	1.00
	Allantoin	0.10	0.10
	Glycerin	3.00	3.00
	Disodium EDTA	0.02	0.02
C	Polysorbate 60	0.17	0.17
	Sorbitan Sesquioleate	0.08	0.08
	Stearic acid	1.00	1.00
	Glyceryl Stearate/PEG-100 Stearate	1.00	1.00
	Cetearyl Alcohol	1.00	1.00
	Glyceryl Stearate	0.40	0.40
	Octyldodecanol	0.20	0.20
	Dimethicone	0.50	0.50
	Caprylic and Capric Triglyceride	4.00	4.00
	Cetyl Ethylhexanoate	3.00	3.00
	Mango Butter	0.50	0.50
	Shea Butter	0.50	0.50
D	Triethanolamine	0.15	0.15
	Water	1.00	1.00
E	Phenonip	1.00	1.00
F	Andrographis Paniculata Extract	0.00	2.00

The exclusion criteria included the following:

- The subject was pregnant or nursing (or planned to become pregnant within six months)
- The subject received immune-suppression treatments within one month prior to this study
- The subject participated in a previous study without waiting the appropriate intervening period of three months between studies
- The subject had sensitive or hypersensitive skin
- The skin around the test site was damaged (sunburn, tattooing, scars or other disfigurement)
- The subject used a similar treatment within three months prior to this study
- The subject had a chronic disease (including diabetes, asthma of high blood pressure)

Each volunteer represented their own placebo, applying either the placebo or the test emulsion on each half of the face twice daily (in the morning and evening). Test sites of all subjects were divided into two groups (A group: Placebo, B group: Test Product) according to the block randomization. An oil-in-water emulsion was used (formulation shown in Table 1).

#### 3.9.2. Measurement of Skin Hydration, Using the Capacitance Method

The skin hydration was measured using a Corneometer^®^CM 825 (Courage + Khazaka, Cologne, Germany). In this study, the skin hydration on the cheek was measured three times, and the average of the measurement values was taken before the treatment, and at four and eight weeks after the treatment.

#### 3.9.3. Measurement of Skin Wrinkling, Using Three-Dimensional Imaging

Skin wrinkling was were evaluated with a 3D skin imaging system (PRIMOS^®^Premium, GFMesstechnik GmbH, Teltow, Germany) that had been used to measure the 3D-profile of the human skin *in vivo*. In this study, the wrinkling at the crow’s feet were evaluated five parameters (Ra: The arithmetic average value of the profile peaks within the total measuring length; Rmax: The maximum of all peak-to-valley values- Rt- measured over the assessment length; Rz: The average maximum height of the profile; Rp: The maximum profile peak height; Rv: The maximum profile valley height) before the treatment, and at four and eight weeks after the treatment.

#### 3.9.4. Measurement of Skin Sagging, Using Contour Line Image

Skin sagging (lifting) was evaluated using Moire topography. The Moire pattern on cheek was determined by F-ray^®^ (Be Young, Beyuong, Korea) before the treatment, and at four and eight weeks after the treatment; the skin sagging was analyzed using Image-pro^®^ plus (MediaCybernetics, Warrendale, PA, USA).

#### 3.9.5. Measurement of Dermal Density, Using an Ultrasonic System

The dermal density was evaluated using a Dermascan^®^ C 20MHz (Corex, Hadsund, Denmark). In this study, the dermal density on the cheek was analyzed for intensity (%), using special software (B-scan mode) before the treatment, and at four and eight weeks after the treatment.

#### 3.9.6. Statistical Analysis of the Clinical Study

A statistical analysis was conducted with SPSS^®^ software (IBM, Armonk, NY, USA). In this study, to compare between the time points, or between the A and B groups, a statistical analysis of the variables for parametrics was conducted using the RM ANOVA. A statistically significant difference was set at *p* < 0.05. The rate of decrement (%) or increment (%) is defined as: (Before treatment − After treatment)/Before treatment × 100.

## 4. Conclusions

Our findings revealed that the APE promotes proliferation of EpSCs through up-regulation of integrin β1 and VEGF expression. The VEGF might affect collagen synthesis of NHFs as a paracrine factor. Clinical studies further suggested that treatment with formulations containing APE confers anti-aging benefits.
